# A Review on Alkali-Silica Reaction Evolution in Recycled Aggregate Concrete

**DOI:** 10.3390/ma13112625

**Published:** 2020-06-09

**Authors:** Miguel Barreto Santos, Jorge De Brito, António Santos Silva

**Affiliations:** 1 School of Technology and Management, Polytechnic of Leiria, Campus 2—Morro do Lena—Alto do Vieiro, 2411-901 Leiria, Portugal; miguel.santos@ipleiria.pt; 2 CERIS, Instituto Superior Técnico, University of Lisbon, Av. Rovisco Pais, 1-1049-001 Lisbon, Portugal; 3 National Laboratory for Civil Engineering, Av. do Brasil 101, 1700-066 Lisbon, Portugal; ssilva@lnec.pt

**Keywords:** alkali-silica reaction, recycled aggregate, concrete degradation, test-methods, characterization

## Abstract

Alkali-silica reaction (ASR) is one of the major degradation causes of concrete. This highly deleterious reaction has aroused the attention of researchers, in order to develop methodologies for its prevention and mitigation, but despite the efforts made, there is still no efficient cure to control its expansive consequences. The incorporation of recycled aggregates in concrete raises several ASR issues, mainly due to the difficult control of the source concrete reactivity level and the lack of knowledge on ASR’s evolution in new recycled aggregate concrete. This paper reviews several research works on ASR in concrete with recycled aggregates, and the main findings are presented in order to contribute to the knowledge and discussion of ASR in recycled aggregate concrete. It has been observed that age, exposure conditions, crushing and the heterogeneity source can influence the alkalis and reactive silica contents in the recycled aggregates. The use of low contents of highly reactive recycled aggregates as a replacement for natural aggregates can be done without an increase in expansion of concrete. ASR expansion tests and ASR mitigation measures need to be further researched to incorporate a higher content of recycled aggregates.

## 1. Introduction

Concrete is produced with materials extracted from nature and, therefore, the use of non-renewable natural resources for its manufacture has, over the years, become a sustainability problem. The reduction of natural resources with the minimum characteristics required to not compromise the durability and performance of concrete structures is a current concern of the technical and scientific community. On the other hand, the construction and rehabilitation of structures produces waste as well as the demolition of some older structures. This cycle will continue over the years due to the finite useful life of the structures; therefore, it is necessary to ensure an effective and sustainable destination for the construction and demolition waste.

Reuse of construction and demolition waste (CDW) began at a large scale after the end of World War II, with large quantities of concrete rubble becoming available and an increased need for new aggregates for reconstruction of damaged or destroyed structures [1].

CDW recycling is one of the guiding strategies of today’s sustainable culture. The use of recycled aggregates (RA) from concrete structures on a new concrete mix is pointed out as a contribution to sustainable construction criteria. However, RA constitution and behaviour on concrete is different from natural aggregates (NA). In this article, more emphasis will be given to coarse RA.

**Figure 1:**
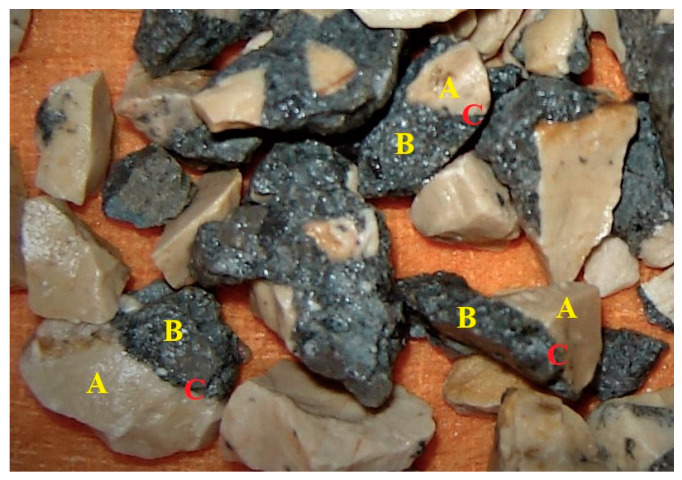
RA constitution. (**A**) Coarse NA; (**B**) Adhered mortar; (**C**) Interface between coarse NA and adhered mortar.

Coarse RA consists of coarse NA and adhered mortar or cement paste. The adhered mortar consists of the cement matrix (composed by the interfacial transition zone (ITZ) between the aggregates and the cement paste, and by the cement paste itself) and fine NA [2,3]. Figure 1 exemplifies coarse RA’s constitution.

The replacement of NA with RA in concrete causes several changes in the fresh and hardened properties of concrete due to RA’s characteristics. Besides, depending on their properties and replacement ratios, the use of RA in concrete requires some additional attention to achieve acceptable properties. Concrete performance is frequently inferior in recycled aggregate concrete (RAC) than in natural aggregate concrete (NAC), but the variation of its properties is similar to that of NAC [4]. Therefore, it is feasible to control and recommend its use with normative regulations.

The incorporation of RA in concrete leads to questions about its durability, including its behaviour related to alkali-silica reaction (ASR), which is one of the major causes of concrete degradation. ASR occurs in concrete as a result of reaction between alkaline pore solution and reactive silica forms within the aggregates [5]. The reaction develops a gel that expands by water uptake and causes concrete cracking [6,7,8]. ASR gel expansion increases the internal stresses of concrete and contributes to decrease its modulus of elasticity, flexural strength, and compressive strength, and activates/intensifies other concrete degradation processes [6,9,10,11,12,13].

Assuming that ASR can occur in RAC and its event may be a consequence of the natural aggregates’ reactivity also present in the RAC, it is important to understand when and why the replacement (partial or total) of NA (reactive or non-reactive) with RA becomes harmful to the new concrete.

This paper reviews the main findings on ASR development in RAC.

## 2. Research Methodology

The main objective of this research is to create a study basis on ASR in RAC, so that the research that currently exists on NAC can be similarly pursued on RAC. Considering this intention, a research methodology was outlined.

First, a list of the principal publications about RA and RAC was collected in order to summarize recent knowledge on this material. Research results were organized, and the main findings were organized and briefly described. Summarized comments on RA and RAC presented on this item are mainly qualitative, comparing the main properties with conventional and well-known properties of NA and NAC.

ASR problem in conventional concrete is presented in Section 4, with focus on aggregate reactivity evaluation, ASR diagnosis and prognosis, the prevention and mitigation, as well as the impact on NAC. The research attempts to indicate the most recent knowledge on the expansive reaction issue, indicating the current reference manuals, methodologies, tests, and threshold limits on ASR study.

Afterwards, considering the properties of the RA and ASR problem in conventional concrete, the main doubts and questions about the possible development and impact of ASR in RAC were outlined and answers were sought, as summarized in Section 5. In this review, the lack of information about RA’s reactivity is evident and articles about ASR in RAC are also reduced. Taking this into consideration, the review strategy had to be more comprehensive and considered the following types of publication to be reliable: articles in the Scientific Citation Index; articles in conference proceedings; Doctoral and Master thesis; published technical reports.

For each publication, an analysis was made in order to establish a conductive line of research. The use of different aggregate types, cement, mix design, aging or test performed hindered the collective analysis. The option was that each publication was individually assessed, highlighting the commonalities on main results and conclusions. Several doubts are still without answer and compromise the development of specifications for the use of RAC, promoting a sustainable future in construction.

## 3. Properties of Concrete with Recycled Aggregates

This section summarizes the main concepts on RA and RAC. The most important consequences of the total or partial incorporation of coarse RA in concrete are highlighted. The RA properties are mainly conditioned by the source concrete’s properties, which include the type of natural aggregate, the ITZ and the adhered mortar quality. The adhered mortar is considered the main cause of the RA properties’ decrease.

Coarse RA have higher porosity and water absorption than coarse NA of similar origin, caused by the adhered mortar. RA can absorb 3–12% of water, depending on whether it is coarse or fine and its old concrete characteristics [3,14,15]. RA also show a lower density, bulk density, crushing, and abrasion resistance, usually depending on the content and quality of the adhered mortar [16,17,18]. Juan & Gutiérrez [19] state that only coarse RA obtained from a source concrete with a minimum of 25 MPa of compressive strength and a maximum of 44% of adhered mortar should be used.

Concrete’s physical characteristics and the presence of anhydrous cement particles in RA are two opposing mechanisms that, according to Katz [15], can affect the properties of RAC, together with the characteristics of the new cement matrix. The properties of RA have an impact on the properties of RAC that are fairly discussed in the literature [3,15,16,17,20,21,22,23,24,25,26,27,28,29,30,31,32,33,34,35,36,37,38,39,40,41,42,43], leading to some summary conclusions:

- The porosity of coarse RA negatively influences the compressive strength and modulus of elasticity, tensile strength, abrasion resistance, shrinkage, water absorption, carbonation resistance, and chloride ion penetration. The effect of RA depends on the property under analysis and on the mechanical strength of the RAC to be produced;
- There are no marked differences between RAC and NAC, if the quality of RA is good, especially in the properties that are more dependent on the new ITZ, such as compressive strength, splitting tensile strength and abrasion;
- The microstructure characteristics of the ITZ of coarse RA are different from those of the ITZ of coarse NA and are influenced by the original concrete (OC)’s properties;
- RAC fracture can occur through RA and not necessarily through the new ITZ. Hence, the adhered paste may be the weak point when its strength is less than that of the new ITZ;
- There is a strong bond between the coarse RA and the surrounding paste in RAC probably due to the angularity of coarse RA and a residual cohesive effect on its surface;
- The modulus of elasticity, shrinkage, water absorption, and properties related with durability are more dependent on the adhered mortar’s characteristics in RA. So, it is more difficult to maintain these properties in RAC equal to those of a reference NAC, although there are interesting results of concrete properties with RA of good quality.

## 4. ASR Problem on Concrete

ASR is an issue still gaining importance in NAC [44,45,46], although there are already preventive recommendations to avoid ASR occurrence in new concrete constructions [47]. These recommendations include limiting the alkalinity of the concrete’s pore solution, ensuring the use of non-reactive aggregate combinations, reducing moisture access or maintaining the concrete sufficiently dry, and also modifying the properties of the gel to be non-expansive [47]. However, despite advances in prevention, new cases of affected structures continue to be discovered around the world.

ASR occurs between unstable reactive silica constituents that exist in some aggregates and the hydroxyl ions (OH-) and alkali ions (Na+, K+) present in the interstitial binder solution. Hydroxyl ions initially attack the silanol group (Si–OH) releasing water and attracting the alkali cations in the middle. The siloxane group (Si–O–Si) is then attacked, in a second stage, by hydroxyl ions, fragmenting the bonds of the group and replacing them with silicates. Solution silicates reacts with calcium to form calcium and aluminium silicate hydrates (C–S–H and C-A-S-H) and ASR gel [48,49,50]. Several discussions concerning the fundamentals of ASR mechanism were analysed and summarized in a Figueira et al. [46] review.

Methodologies for ASR diagnosis [12,51,52,53,54,55] are essential to identify and confirm its presence in concrete. However, it is important that the aggregates’ reactivity is properly detected prior to its use, since ASR development is generally slow and is observed only several years after the structure is in service.

RILEM published in 2016 a compilation reviewing and updating the methodologies and recommendations for ASR prevention and mitigation in NAC. The test-methods to evaluate the potential alkali reactivity of aggregates for use in mortar or concrete are: the petrographic examination method according to RILEM AAR-1 [47], similar to the ASTM C295 standard [56]; the accelerated mortar-bar test method (AMBT) according to RILEM AAR-2 [47], similar to ASTM C1260 standard [57]; the concrete prism test (CPT) at 38 °C according to RILEM AAR-3 [47], similar to ASTM C1293 standard [58]; and the concrete prism test (CPT) at 60 °C according to RILEM AAR-4 [47], similar to AFNOR P 18-454 standard [59].

In the AMBT, the aggregate is considered potentially deleterious if the average expansion of the mortar bar after 14 days of immersion in NaOH at 80 °C is higher than 0.20%. For expansion values within 0.10–0.20% at 14 days, it is recommended that the test proceeds until 28 days, the aggregate’s reactivity being considered doubtful when the expansion at 28 days is lower than 0.20%. Expansions of less than 0.10 % at 14 days after immersion are indicative of innocuous behaviour in most cases. In the CPT methods, the composition in evaluation is considered non-expansive if the average length of the prisms conserved in a moist environment at 38 °C is below 0.05% at 1 year or 0.02% at 12–15 weeks when conserved at 60 °C.

The long duration of the tests and the aggressive environment used to accelerate the ASR development can compromise the prompt assessment of aggregate reactivity. Several suggestions for tests modifications or new analysis procedures are present in literature, such as the use of a CPT test at 38 °C with prims that immerged in an alkaline solution [60] or the application of a stiffness damage test for assessing the depreciation of concrete mechanical properties [61].

Cement is one of the main sources of alkalis supply, although any other source of alkalis can be mobilized for ASR development. The equivalent sodium oxide content (Na_2_O_eq_) is conventionally used to indicate the alkali content of Portland cement and is usually limited to values less than 0.60% to mitigate ASR. Nowadays, it is more recommended to compute all alkalis available in concrete constituents instead of only the alkalis content of the cement, and the concrete Na_2_O_eq_ is generally limited to a maximum of 2.5 kg/m^3^.

In terms of ASR prognosis, the knowledge about the residual expansion and the soluble alkalis content is of the uttermost importance [62]. It is known that the ASR expansion increases with the alkali enrichment of cement paste with ageing, which is mainly attributed to the alkalis released by the aggregates [63]. The evaluation of the alkalis released by the aggregates is usually determined by laboratory extraction tests, where the aggregates are immersed in alkaline solutions [47,62]. Recently, Berra [64] suggested an autoclave method to minimize leaching and increase the alkalis extraction in a short testing period.

## 5. Research Issues and Concerns about ASR Development on Concrete with RA

In general, standards regard RA as a potentially reactive aggregate. When RA of concrete are intended to be used, EN 12620 [65] states that it will be necessary to confirm that the OC does not contain potentially reactive aggregates, and when the alkalis content of the new concrete (or cement) is limited, the alkalis content of recycled concrete aggregates should be determined and taken into account.

The use of the same prevention and evaluation methodology on RA reactivity is very restrictive since it is quite difficult to control, analyse and limit the origin of RA, and thus, the reactive silica present in the fine and coarse original NA. The amount of alkalis content of the adhered mortar present in a RA structure is also laborious to control. Age, exposure conditions of the old concrete before it is recycled, and the heterogeneity of RA have influence on its reactivity.

However, in addition, other differences in ASR development can occur either due to the properties of the RA or of the RAC itself. It is not certain that all RA have a negative influence on the ASR development, in particular when their reactivity has been extinguished or is reduced. Also, the characteristics of RAC are distinct from those of NAC, which increases doubts about its behaviour in relation to ASR. Research about ASR in RAC exists but is still very preliminary, and there are still several doubts about the influence of RA from concrete on ASR development. Considering previous reasoning, the main research issues and concerns about ASR development on RAC are:

- Are the existing reactivity tests sufficient and adequate to study RA?
- Can an increase in alkalis content of a new concrete mix or an increase of fresh reactive aggregate surfaces originated during the RA’s preparation make a RA reactive?
- Can a concrete with natural reactive aggregate that has not been exposed to the optimal humidity conditions for ASR development become reactive in a new RAC?
- Since the porosity is higher in RAC than in NAC, can this characteristic benefit the ASR gel accommodation and counteract its expansion?
- Can a long exposure of RA to alkalis decrease or extinguish its potential reactivity or, on the contrary, can it be reactivated in contact with new cement?
- Has the ASR gel formed in RAC different properties than in NAC?
- Are the properties of RAC and NAC different when ASR occurs?

To answer these questions, a critical literature review and analysis was made. Section 6 separates the articles by themes and focuses on the main objectives of each research, the common parameters and the main conclusions obtained, as well as providing a discussion.

## 6. ASR in Concrete with Potential Reactive RA

### 6.1. Suitability of ASR Accelerated Tests to Evaluate RA Reactivity

The adhered mortar in RA increases the porosity of the aggregate and can accommodate some of the ASR gel formed, being able to “hide” or “delay” the expansion. Crushing and washing the mortar during sample preparation for the accelerated tests may also change the properties of the RA or the sample under study. The heterogeneity that may exist in RA with different origins can make this assessment difficult. Also, the high duration of tests is still a concern when RA are used. Several proposals of modified tests are presented in the literature to overcome these difficulties, and some are summarized next.

Gress & Kozikowski [66] propose the acceleration of AMBT and CPT ASTM test-methods through an increase in temperature, microwave energy applications, ultrasonic energy utilization, and alkali boosting. The authors also proposed to increase the size of AMBT test specimens to 76.2 × 76.2 × 279 mm^3^ when RA are used. They also used different test specimens: larger prisms; prisms with lateral holes to facilitate the NaOH access; solid cubes; and cubes with lateral holes. The changes introduced in the test-methods procedures were effective in ASR acceleration.

Scott IV [67] states that ASTM C 1260 test for RA crushes and destroys the aggregate matrix, which causes separation of the aggregate from the mortar, thus not testing RA as a whole. The author considered that ASR tests should be focused on RA’s absorption capacity, mortar fraction and free alkalis content. His proposal is to replace the test specimens used in the mortar-bar test with others with the size of the concrete prisms used in the ASTM C 1293 test, but applying ASTM C 1260 test conditions. Furthermore, the limit expansion value at 28 days was decreased from 0.10 to 0.04%. Scott IV [67] used altered samples, based on the work of Gress and Kozikowski [66], but vacuum sealed using a plastic bag. Considering that cathodic protection of reinforced concrete promotes ASR, Scott IV [67] also studied a method to accelerate the reaction through an electric current.

Adams et al. [68] considered that aggregate washing, used in NA AMBT and CPT tests, can reduce adhered cement paste in the RA, promote the hydration of anhydrous cement particles in RA, and/or leach calcium or alkalis from aggregates and adhered paste. The authors proposed limited washing at time intervals for each of the size fractions. The authors analysed four types of RA from NAC blocks that were under natural exposure over a long period in a CANMET study [69]. The blocks were selected by age, extent of ASR damage and mineralogy of reactive NA.

Adams et al. [68] also modified the mixing process because of the water absorption of RA. First they immersed the RA in water for 30 min and reached 85% of RA saturation. After this period, extended for another 30 s in mixing, they inserted cement and continued mixing. The results showed that AMBT can be used to evaluate ASR in RAC as long as precautions are taken in the mix.

Johnson & Shehata [70] proposed the use of concrete micro-bar test (CMBT) based on the RILEM AAR-5 test method [47], to minimize RA characteristics modification and compare results with those of the AMBT. They used RA from the same source used by Adams et al. [68]. The required fractions for AMBT were produced using large and small jaw crushers as well as a disk pulveriser. After crushing, RA were washed based on the Adams et al. [68] method. In CMBT, the batch water was corrected for aggregate absorption during mixing and the alkalis were boosted until 1.5% Na_2_O_eq_ in some mixes. After 24 h curing, the samples were soaked in water at 80 °C for another 24 h and then immersed in a 1 N NaOH solution at 80 °C. The authors believe that AMBT and CMBT can be used to evaluate the reactivity of RA if high absorption of the RA is considered and a representative sample of the crushed materials is used (maintaining the relative proportion of residual mortar-to-stone). Avoiding several crushing steps to achieve sand size is indicated as the main advantage of CMBT, because the adhered mortar and NA are represented in the same proportion and condition as they are in concrete. CMBT was effective in evaluating the reactivity of RA and NA when an expansion limit of 0.04% at 28 days or 0.10% at 56 days was used. For AMBT, the authors propose to process the RA with a jaw crusher followed by a disk pulveriser, which was found to produce a fine aggregate size distribution that better represents the reactivity of the coarse RA. Johnson & Shehata [70] observed that washing RA slightly reduces the expansion in AMBT, while changes in the crushing method were more significant in the expansion.

To study the influence of the water absorption of RA on ASR tests, Delobel et al. [71] studied two standard experimental procedures using mortars and microbars, which were evaluated according to the NF P18-594 test-method. The authors used two industrial coarse RA incorporating a siliceous limestone aggregate and a mortar rich in alkalis. They tested three protocols to consider the water absorption of RA during mortar manufacture: using dried RA without taking into account the water absorption; using dried RA and the water corresponding to absorption added to the bowl during mixing; pre-saturation of aggregates 96 h before mixing with the water absorption value +5%. Although all results were positive for ASR, the authors observed a higher expansion on AMBT when they did not take into account the water absorption because, according to them, as soon as the mixing starts, part of the mixing water is absorbed by the aggregates, reducing the quantity of effective water, which reduces the workability of the mortar and the effective water–cement ratio of the in-place material, leading to a reduction in the matrix porosity and probably a larger expansion of material caused by expansive products.

Beauchemin et al. [72] evaluated CPT to assess the potential ASR reactivity of RA. The authors used reactive coarse RA from concrete blocks made with a variety of reactive coarse aggregates from CANMET [69] and coarse RA from three different structural elements of a demolished concrete overpass (foundations; bridge deck; columns) affected by ASR. The residual mortar content of the RA was also analysed. CPT was made according to CSA A23.2-14A. The authors produced several series of concrete with replacement ratios of 25, 50 and 100% of coarse RA with changes in adhered mortar content and RA’s moisture conditions.

Beauchemin et al. [72] recommended using aggregates under a saturated state. The authors considered that using in the mix process an additional water content will increase the risk of segregation. Because of the variability of adhered mortar content with RA size, the authors also suggested following ASTM C1293 by using the dry-rodded bulk density to determine the aggregate/sand ratio, instead of the fixed 60:40 ratio of CSA A23.2-14A.

### 6.2. Potential Influence of RA Recycling Process on ASR Development

Exposure of fresh faces of the original reactive NA resulting from the RA crushing seems to increase RA’s reactivity, getting worse with multiple cycles. Still, adhered mortar and aggregate only with mortar can also underestimate or attenuate the ability to expansion. There are certainties on research about the recycling effect on RA cracking and NA exposure, but doubts exist on its effect on expansion due to the aggregate reactivity level and to the adhered mortar damping.

**Figure 2:**
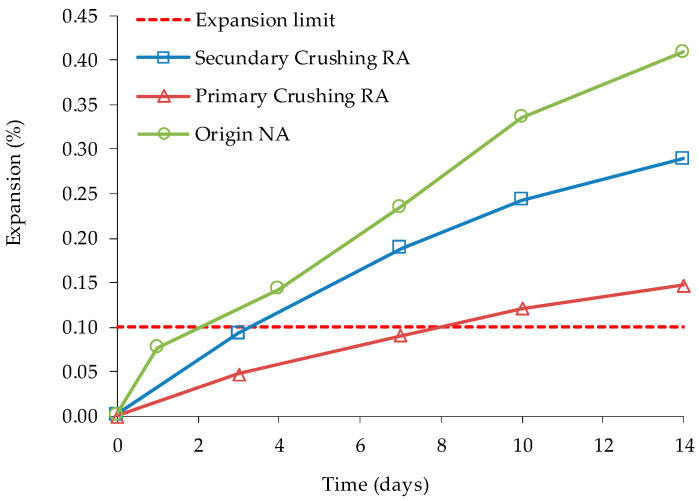
Expansion of RA from primary and secondary crushing in the ASTM C 1260 test, adapted from [73].

Shehata et al. [73] found that in AMBT the fine fraction produced by primary crushing caused lower expansions than that resulting from secondary crushing of coarse RA (Figure 2). Still, the authors also noted that it is possible that processing of coarse RA to reduce the particles size for AMBT may produce samples with less reactive aggregate and more mortar than in the original coarse RA, which will underestimate the coarse RA’s reactivity. The authors used concrete with ASR that was for 12 years under natural exposure according to ASTM C 1260 and CSA A23.2-14A procedures. The authors varied the alkali content of the compositions and the content of mineral additions and lithium nitrate. In addition to fresh new faces, Shehata et al. [73] stated that high RAC expansions can be attributed to a significant increase in alkali contribution by residual mortar in RA and/or expansion of the ASR gel in RA when exposed to a high level of moisture in the new concrete.

Adams et al. [68] found that the increment in RA crushing, as also found by Shehata et al. [73], leads to higher access to reactive NA and, consequently, higher expansions. RA’s reactivity will depend on the amount of reactive NA present in the RA and also on the replacement ratio of NA with RA.

Beauchemin et al. [72] found that the reactivity/expansion of the OC of RA will influence the expansion of RA. The author’s results show that RA with a high degree of reactivity/expansion/damage can undergo less expansion than concrete with a similar content of unreacted aggregate material of the same origin. The authors attributed this fact to the consumption of reactive phases of RA and to the fact that RA have adhered mortar that limits the tendency of NA to react. However, aggregate crushing also causes the exposure of new fresh aggregate surfaces that may increase reactivity, as Shehata et al. [73] also mentioned.

### 6.3. Potential Influence of RA Alkalis Content on ASR Development

The increase in concrete alkalis’ content due to alkalis present in RA’s adhered mortar and in new cement used in a RAC mix is a concern to ASR development. Stark [74] states that the ASR expansion regulator in an OC of the RA was the alkalis content of the interstitial solution and that the re-introduction of alkalis through the new cement in a RAC restarted ASR. Moreover, Stark [74] suspected that ASR is more dependent on the alkalis content of the new cement of RAC mix than on that of the old concrete.

**Figure 3:**
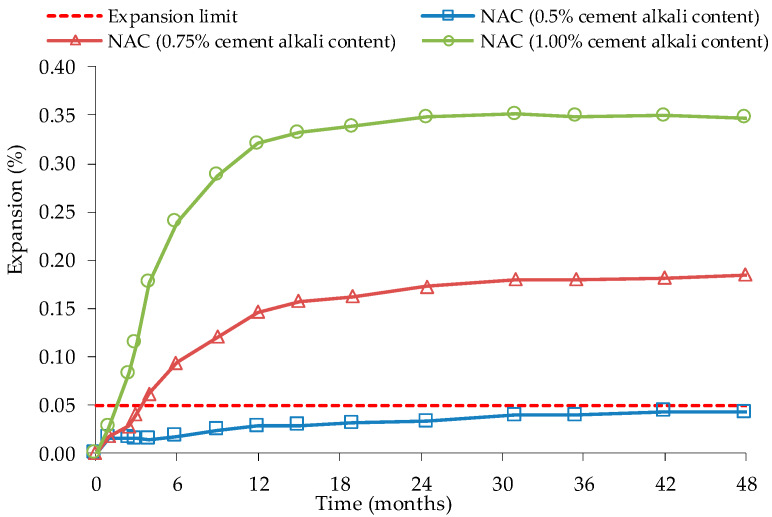
Expansion of NA with cements of different alkalis content in ASTM C227, adapted from [74].

**Figure 4:**
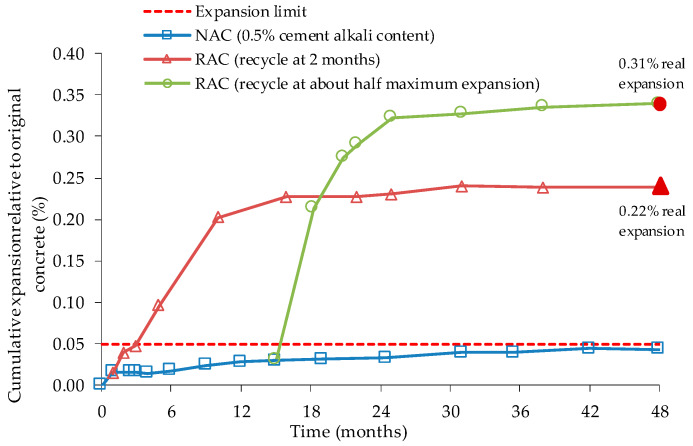
Expansion of high alkali mixes with coarse RA, from low alkali OC, and high alkali cement in ASTM C 227, adapted from [74].

**Figure 5:**
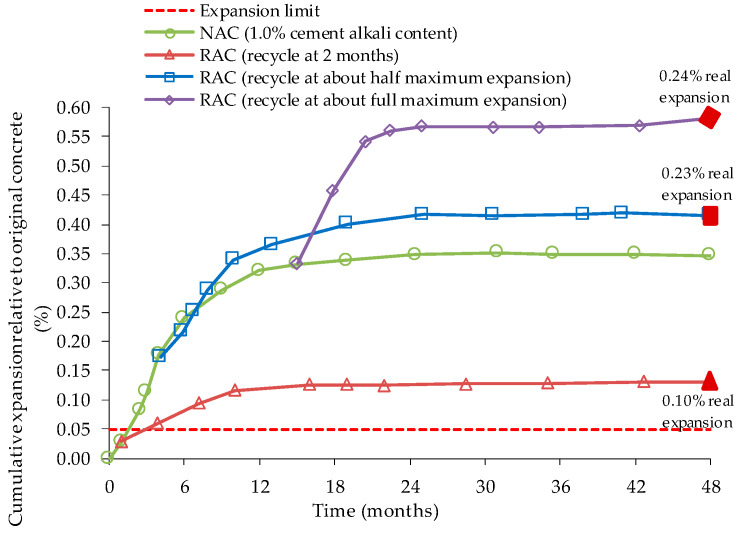
Expansion of high alkali mixes with coarse RA, from high alkali OC, and high alkali cement in ASTM C 227, adapted from [74].

In a Stark’s [74] study, coarse and fine potentially reactive aggregates were tested with Portland cement with 0.50, 0.75 and 1.00% of Na_2_O_eq_, which were afterwards exposed for 48 months to ASTM C 227 test conditions (Figure 3). The samples were removed after 2 months of testing, at half and at the maximum expansion level. The author states that no washing of the RA was done to avoid losing characteristics, mainly to preserve alkalis content in the pore solution in the OC and thus also in the RA used. After crushing, concrete expansion tests were made with high alkali RAC of coarse RA from low alkali OC (Figure 4) or high alkali OC (Figure 5), and high alkali cement. Innocuous fine NA was used. All RAC expansion was higher than OC expansion, boosted by OC alkalis content of the interstitial solution. Stark [74] also observed that the largest expansion occurred in RAC with RA produced with 1.00% of Na_2_O_eq_ and crushed at its maximum expansion value in ASTM C 227. Although reactive NA does not react in concrete with low alkali cement, the reactivity of NA remains after crushing. It was also observed that, in some cases, ASR leads to excessive expansions when cement with high alkali content is used mixed with RA from an OC with low alkali content.

Gress & Kozikowski [66] results, with modified AMBT, shows that RA exhibits a smaller expansion than NA when using cement with 1.15% of Na_2_O_eq_. The authors justified the results with the lower reactivity of RA per unit volume and its higher porosity to accommodate ASR gel. RAC expand less than NAC, although the opposite occurs when alkalis’ content increases.

Scott IV & Gress [75] found initial expansion in RAC. The authors state that the paste fraction of RA is responsible for the expansion. According to the authors, the high absorption allowed water to enter the paste fraction of the RA of concrete. This caused expansion in concrete during early hydration, when the new matrix was soft.

Dhir et al. [76] examined the problem of ASR when using RA from several origins and observed that all aggregates studied achieved values in AMBT below the expansion limit of 0.10% at 14 days. Regarding alkalis release, the highest values were obtained from bricks and concrete blocks, with Na_2_O_eq_ release values, by mass, between 0.47 and 0.61%. Concrete from demolition and concrete produced in laboratory released about 0.15 and 0.20% of Na_2_O_eq_ by mass, respectively.

Dhir et al.’s [76] study consists of evaluating aggregates’ reactivity and alkalis release from CDW using ASTM C 1260 procedure and the alkalis CANMET extraction test-method [77]. They tested materials produced recently and from demolitions with a cement Na_2_O_eq_ of 0.6%, making different RA groups (lightweight concrete, brick, plasters, floor-regulating aggregates, among others). The authors also produced concrete with a high and normal reactivity, some with mineral additions (fly ash or granulated-blast furnace slag) and compressive strength between 25 and 50 MPa, crushed at 28 days. Some of the materials were subjected to CPT, according to BS 812-123, similar to RILEM AAR-4. Concrete samples were manufactured with a high alkali cement and fine and coarse RA, from RA groups mentioned above, and then combined with a low coarse or fine reactive natural aggregate. After curing, the concrete samples were conditioned in a moist cabinet at 60 °C for 12 weeks. With the exception of concrete samples with RA from the road planning’s, which reached high expansion values, the remaining concrete samples showed low reactivity and little risk of ASR development.

**Figure 6:**
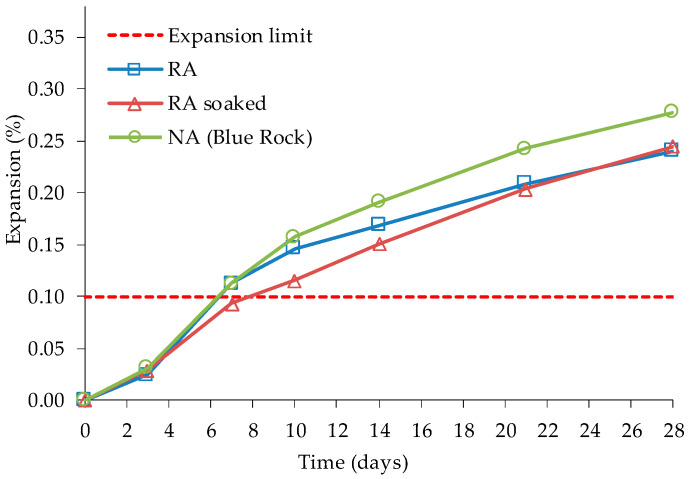
Expansion of saturated and unsaturated NA and RA in the ASTM C 1260 test, adapted from [78].

**Figure 7:**
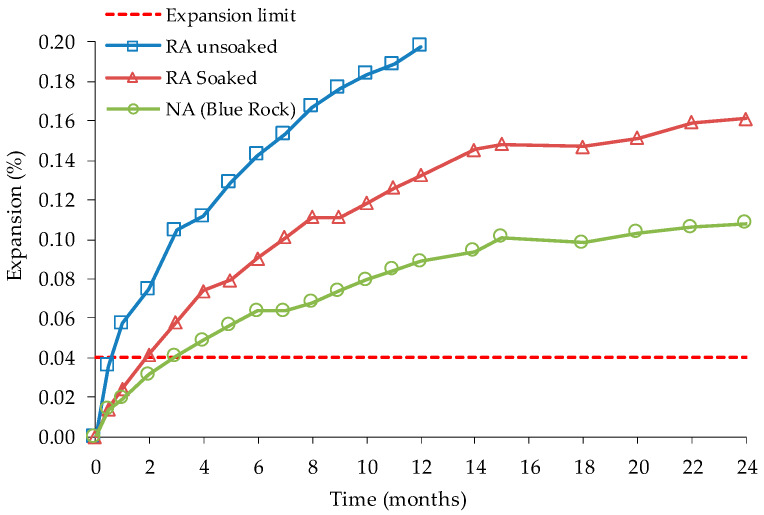
Expansion of saturated and unsaturated NA and RA in the ASTM C 1293 test, adapted from [78].

Li [78] stated that the RA should contribute with alkalis to the cement paste system since it has soluble alkalis in the old paste. However, the interstitial solution does not show the effect of this increase; so the author argues that the interstitial solution is fundamentally controlled by the alkalis content of the cement and the alkalis present in the RA can be in balance with the alkalis in the pore solution. RA used by Li [78] was obtained from recycling a concrete pavement that had been affected by ASR. ASR was caused by a coarse quartzite/quartz-biotite natural aggregate designated by “Blue Rock”, usually used as an ASR reference aggregate. To compare the behaviour of RA and reference NA, the researcher used hydrochloric acid to dissolve the cement paste adhered to the RA and tested them with ASTM C 1260 procedure. Li [78] used vacuum saturation of the RA in the AMBT and CPT ASTM tests. Cements with high alkali content (1.09% Na_2_O_eq_) and, in some cases, one with a low alkali content (0.26% Na_2_O_eq_), were used in the mixes. The author used RA’s saturation to eliminate the effect of cement hydration on the final expansion. However, in AMBT, there was no large variation between saturated and unsaturated RA (Figure 6) probably due to the small size of the particles. In CPT, saturated RAC has larger expansions at the same ages than NAC (Figure 7), as also observed by Scott IV & Gress [75]. Li [78] commented that this can be due to swelling of the old ASR gel in the RA, the reactivation of ASR or the increase in alkali content of the new cement paste.

Scott IV [67] observed a higher content of soluble alkalis in RA, which was attributed to the paste fraction of the RA. Moreover, he observed that RAC had a high initial expansion, which was attributed to the water absorption of the mix during the initial hydration of concrete. Thus, he pre-saturated the RA for 48 h before mixing, filling their pores and assuming that it would eliminate the initial expansions, and used pre-saturation water in the mix, avoiding alkalis loss.

Shehata et al. [73] observed that washing the RA for 18 h in running water did not result in a significant reduction in expansion. The authors considered that it was not enough to clean a large amount of alkalis from the residual adhered mortar of RA and/or that there were sufficient alkalis in the new concrete to reactivate the expansion on the fresh faces of NA present in RA.

### 6.4. Potential Influence of RA Replacement Ratio on ASR Development

The reactivity level of concrete depends on the amount of reactive aggregate used and on its combination with other aggregates. Hence, ASR should not be a problem if only RA with non-reactive NA are used. However, as already mentioned, it is very difficult to control reactivity level/activity of the RA’s source concrete. Studies demonstrate that even using RA with reactive NA, the concrete expansion level depends on the replacement ratio of NA with reactive RA.

The Gottfredsen & Thøgersen’s [79] study showed that the incorporation of 20% coarse RA in new concrete did not cause a critical expansion. The authors observed that the OC was affected by ASR due to reactive constituents in the aggregates’ fine fraction. So, the RAC had a non-reactive coarse NA and a potentially reactive mortar.

Etxeberria [2] studied the microstructural and structural behaviour of RAC [80]. The author used mixes with 0, 25, 50 and 100% of RA from a recycling industry, and with a CEM I 52.5R with Na_2_O_eq_ content higher than 0.6%, keeping 28-day compressive strength constant. The coarse RA’s and adhered mortar’s reactivity were studied separately using the ASTM C 1260 test procedure.

**Figure 8:**
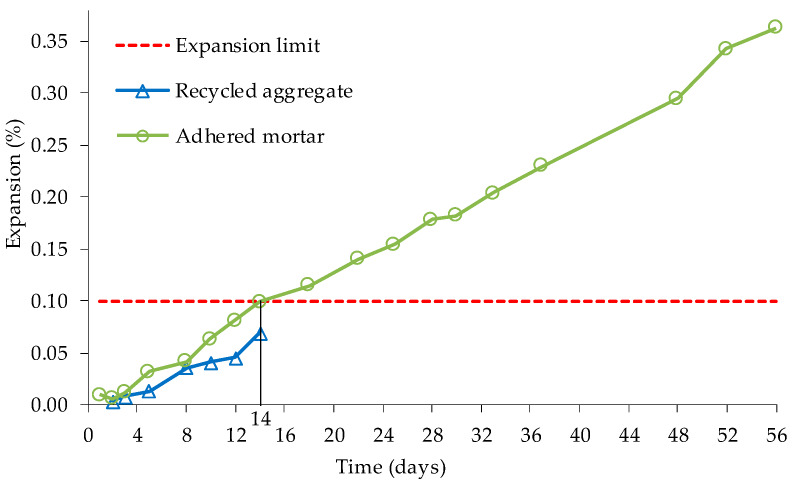
Expansion of recycled aggregate and adhered mortar after 14 days and after 56 days in ASTM C 1260, adapted from [80].

Etxeberria [2] AMBT tests results showed at 14 days an expansion of 0.07% for the coarse RA and 0.10% for the adhered mortar. The tests were extended up to 56 days and the adhered mortar’s expansion increased to 0.19% at 28 days and 0.37% at 56 days (Figure 8). Therefore, the original fine NA present in adhered mortar was suspected to be potentially alkali-reactive. Etxeberria [2] also carried out macroscopic and microscopic tests in concrete specimens in order to confirm ASR evolution. The tests were made after the concrete was stored in a humidity chamber for 8 months at 20 °C and 100% RH. Scanning electron microscope (SEM) analysis shows ASR gel in the interfaces between RA and cement paste, this presence being justified by the increase in the alkalis content of the mix due to the new cement employed (CEM I 52.5 R), originating ITZs with high alkalis content. The interstitial solution reached a high pH, promoting the dissolution of reactive silica on the aggregates’ surface and producing ASR gel. However, Etxeberria did not find losses in RAC’s properties due to ASR, but it may have been because the manufactured beams for structural evaluation were placed in a dry environment.

In Calder & McKenzie’s [81] work, the RA was classified as non-expansive. The authors claim the modification of ASR classification from high to normal reactivity. The authors observed the formation of ASR gel by microscopic analysis. Although ASR gel appeared in some concrete specimens, the authors did not observe significant expansions, even though petrography indicated the presence of reactive silica forms in some RA, that were classified as potentially ASR-reactive.

**Figure 9:**
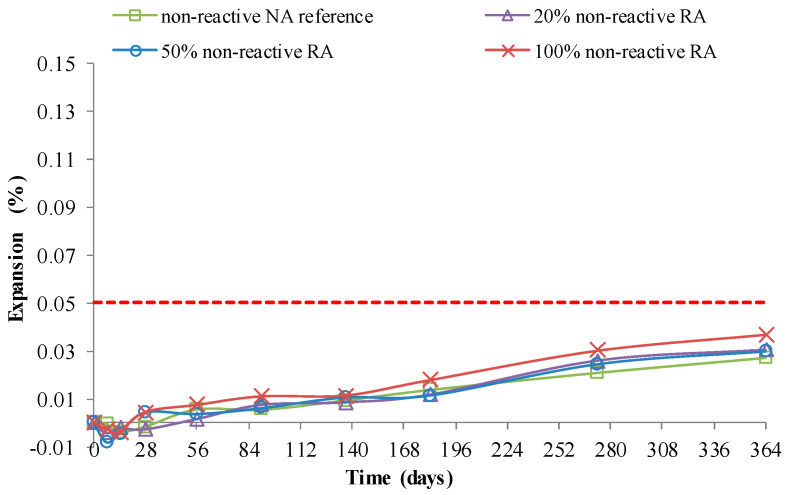
Evolution of the expansion in non-reactive concrete mixes using the RILEM AAR-3 method [82].

**Figure 10:**
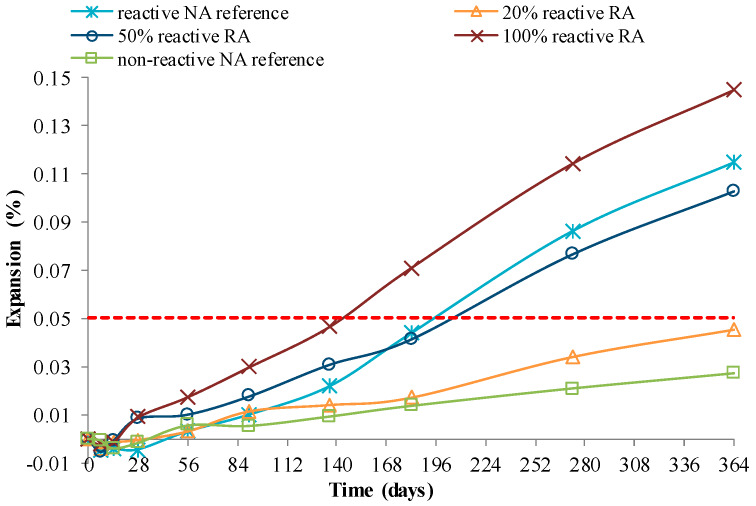
Evolution of the expansion in reactive concrete mixes using the RILEM AAR-3 method [82].

Barreto Santos et al. [82] confirmed that the use of coarse RA with non-reactive NA caused no remarkable expansion in RAC (Figure 9), and observed that, up to 20% of coarse RA as NA replacement, the mix’s reactivity seems not to be significantly affected (Figure 10). The authors also studied the effect of total or partial use of reactive or non-reactive RA on RAC’s behaviour. For this purpose, controlled reactive (full reactive NA) and non-reactive (full non-reactive NA) concrete mixes were used, which were differently aged to simulate old and recent concrete. Before recycling, both types of concrete underwent either accelerated aging (climatic chamber at 38 ± 2 °C and relative humidity higher than 95%) or natural exposure condition. The RA and the coarse NA were both separated in different d_i_/D_i_ sizes, and mixed using a method based on the Faury’s reference curve. The author’s prepared four concrete families with differences in mix reactivity, RA replacement contents (0%, 20%, 50%, and 100%), RA age (according to aging suffered) and cement type (42.5R or 52.5R). The reactivity of aggregates was evaluated using ASTM C1260, and the reactivity of concrete mixes was evaluated according to RILEM AAR-3 CPT. The authors also comment that the age and exposure conditions of concrete will have an influence on the RA’s alkali-reactivity potential.

### 6.5. Potential Influence of RA Age and Exposure Condition on ASR Development

**Figure 11:**
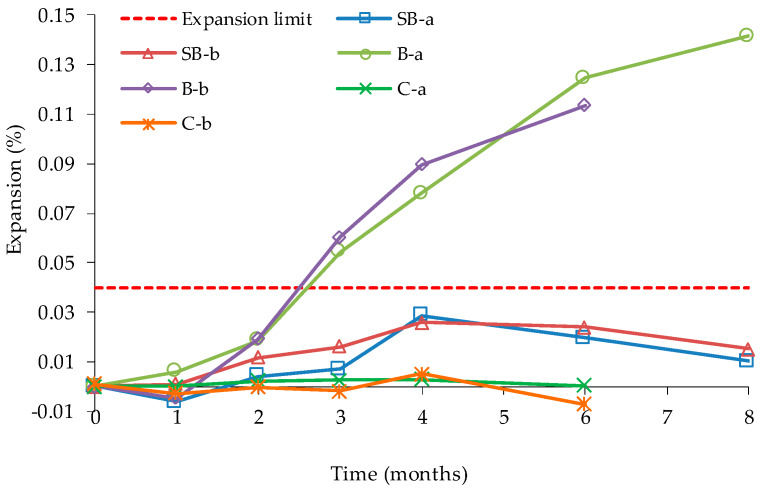
Evolution of expansion of selected aggregates in NF P 18-587, adapted from [83].

The exposure time of a concrete to favourable conditions to ASR development seems to limit the reactivity state of the aggregates and their ability to continue to react after recycling process and incorporation in a new concrete. Desmyter & Blockmans [83] observed that the reactive elements of studied RA preserved only a limited reactivity after years of deleterious ASR in old concrete. The authors analysed a potentially alkali-reactive NA (aggregate B), a non-alkali reactive NA (aggregate C) and a RA (aggregate SB) from the demolition of a bridge affected by ASR. The reactivity of the aggregates was studied by CPT according to the French standard NF P 18-587, similar to the RILEM AAR-3 test-method. Desmyter & Blockmans [83] tests results showed that NA “Aggregate B” (reactive) confirmed to be reactive, while NA “aggregate C” (non-reactive) proved to be non-reactive. RA from the demolition of a bridge affected by ASR, denominated “Aggregate SB” showed an expansive behaviour in the first months, but did not exceed the 0.04% threshold expansion limit at 8 months of the NF P 18-587 standard (Figure 11).

**Figure 12:**
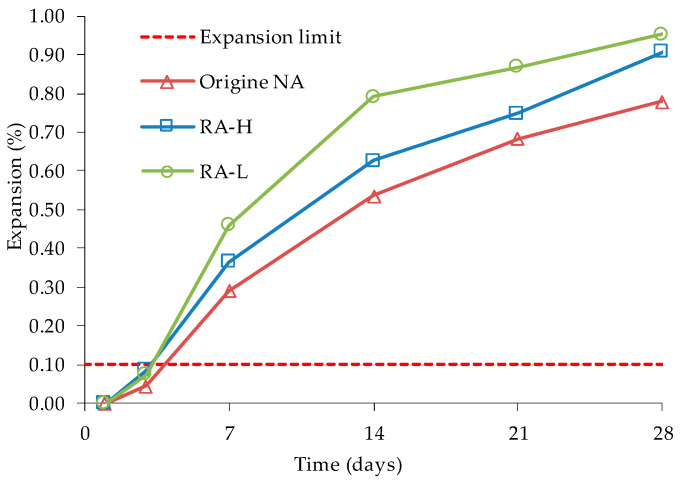
Expansion behaviour of RA and NA according ASTM C1260, adapted from [84].

Nevertheless, Sugiyama et al. [84] found that the existing coarse NA inside the studied coarse RA maintained almost all their reactivity, even after 30 years in the concrete of a bridge affected by ASR. The authors analysed the RA’s reactivity of the columns of a bridge affected by ASR, in Japan. The bridge’s concrete contains a non-reactive river sand and an alkali-reactive andesite coarse aggregate. Two types of RA were produced by crushing, one coming directly from crushing (RA-L) and another that after crushing underwent abrasion to minimize the amount of adhered mortar (RA-H). RA-L was about 60% of adhered mortar and had 6% water absorption, while RA-H had about 30% adhered mortar and had 3% water absorption. All aggregates were classified as alkali-reactive by AMBT (Figure 12). Using CPT, authors claimed that ASR development in concrete with coarse RA was delayed due to the dampening effect of the adhered mortar and the reactive ring around the coarse RA.

### 6.6. Potential Mitigation Measures

There are several procedures, some in use and others still in study, to mitigate ASR in NAC, such as: the use of supplementary cementitious materials (SCM) [85] and lithium-based chemical additions [86,87,88,89].

SCM’s influence the alkalis content in the paste pore solution by reducing the amount of alkalis available for ASR since some were fixed in the reaction with SCM. According to Thomas [85], the reaction that occurs with SCMs is similar to ASR. The reactive silica of SCMs reacts with the alkali-hydroxides, forming ASR gels with a relatively low Ca/Si ratio without expansion behaviour. This author concludes that higher amounts of SCMs are required when there is an increase in the alkalis content of concrete or the potential alkali-reactivity of the aggregates. Silica fume, metakaolin, low-calcium fly ash, and ground granulated blast furnace slags are the most used SCMs.

Lithium-based chemical additions, mainly lithium nitrate, are recognized to be efficient in the prevention and decreasing of the dissolution of reactive silica. However, this mitigation mechanism is not yet fully understood. Tremblay et al. [87] stated that the mechanism that could explain the phenomenon is due to the higher chemical stability of silica in the presence of lithium ions. Kim and Olek [88] stated that Li^+^ facilitates the emergence of a physical barrier on the surface of reactive aggregate and hampers the consumption of alkalis ions. Despite these works, a recent study of Liu et al. [90] on lithium use for ASR mitigation demonstrated that the use of a high concentration of lithium nitrate can be effective for ASR mitigation at the early stages, but after a long period of time, the formation of lithium silicate (Li_2_SiO_3_) occurs and that causes expansion and cracks in concrete.

RAC research also presents proposals for ASR mitigation, with similar measures to those of NAC. Stark [74] recommended the use of low alkali cement and fly ash to inhibit ASR expansion. The author observed that the replacement of cement with 20% of fly ash re-established, in most cases, the levels of safety related to expansion. However, Stark [74] considered that, in order to reduce expansions to values below the limit, without exception, it is necessary to use the combination of low alkali cement and fly ash. Desmyter & Blockmans [83] also observed that the use of low alkali cement or incorporation of granulated-blast furnace slag might improve the durability of RAC, when ASR is a concern.

Sánchez de Juan [91] introduced the ASR problem in RAC during a state-of-the-art analysis on structural concrete design with RA. The author referred to the possibility that RA could potentially be reactive to alkalis, which was justified, according to BRE Digest 433 [92] and Sagoe-Crentsil & Brown [93], by the high alkali content in the adhered mortar. Sánchez de Juan (2004) considered that the use of RA may potentiate ASR in RAC due to the difficulty in monitoring the reactivity of the RA, because of its heterogeneous origin and, as noted by Etxeberria [2], requires separate ASR tests for the characterization of the coarse and fine fractions. Sánchez de Juan [91] found through theoretical calculations that in RAC the use of low alkali cement contents would be necessary, unlike in NAC.

Li [78] states that small amounts of mineral additions or lithium salts should be used to mitigate ASR in concrete formulated with the reference Blue Rock aggregate. The author concluded that 25% of fly ash, or 55% of granulated blast furnace slag or 12% of silica fume, all as cement replacement can also mitigate ASR, but the formation of silica fume clusters can reduce this effectiveness. The author also analysed the mitigation capacity of the use of lithium ions, either by incorporation in the mix or by application in the structure itself. Li [78] found the need for larger amounts of lithium to mitigate ASR in RAC compared to what happens in NAC. In the surface application of lithium nitrate in pavement blocks, a penetration of about 25 mm of lithium ion was observed, with reductions in surface expansion, although, according to the researcher, there was no effect on internal expansion. Li [78] also made an analysis of the interstitial cement paste of mortars produced with the natural Blue Rock aggregate or with RA. The analyses, which were complemented by determining the calcium hydroxide content by thermogravimetry, showed that the mineral additions significantly reduced the contents of calcium hydroxide and alkalis and the pH of the interstitial solution.

Scott IV [67] found that only mixes with low alkali cement, 55% granulated blast furnace slag, 25% fly ash or 100% lithium nitrate relative to the total alkali content were effective in ASR mitigation. Similar to previous researchers, Shehata et al. [73] also observed that RAC suffered similar expansions to the reference NAC, although they noted a need to introduce larger amounts of mineral additions to mitigate ASR.

## 7. Conclusions and Challenges for Future Research

One of the main sustainability challenges in construction is focused on eliminating waste and preserving natural resources. The reuse of construction and demolition waste has become a reality to improve such a purpose. The incorporation of RA in structural concrete to construction intends to promote the sustainability and reduce environmental issues.

However, according to the literature, there are a growing number of structures affected by ASR, which increases the possibility of having potentially reactive RA in the future if recycled. Since RA do not come only from one type of concrete or from one structural element, unlike controlled concrete, it is difficult to measure and control the amount of alkalis present in the adhered mortar of RA or the content of reactive silica present in NA.

Literature results are often contradictory about the effects of RA on ASR occurrence. However, the main conclusions of the analysis of literature, in order to contribute to ASR in RAC’s knowledge and discussion, are the following:

- ASR can continue in RAC, in particular: by increasing the alkali content of the medium; fresh faces of NA contained in the RA because of crushing; a more favourable environmental exposure for ASR development;
- Secondary crushing increases RA reactivity if reactive NA are present. Adhered mortar can limit the tendency of NA to react;
- Age and exposure conditions of recycled origin concrete seem to be reflected on RA reactivity, probably due to the consumption of aggregate reactive phases;
- RA’s mitigating effect on the AMBT and CPT expansion, caused by the adhered mortar, should be considered in future tests, reviewing methodologies and expansion limits;
- The size of test specimens should be increased when RA are used; AMBT and CPT tests should be reviewed to accommodate RA; RA washing limitation and water absorption of RA should be considered;
- AMBT for ASR evaluation, although faster, can cause misleading results due to the fragmentation of RA, which causes the detachment of the adhered mortar, creating fine RA with different characteristics from those of the original coarse RA; our suggestion is to test separately the adhered mortar and NA or that only RA from coarse RA (i.e., from secondary crushing);
- Soluble alkalis content of the RA should not be ignored since they will increase the alkali content of RAC;
- High amounts of mineral additions (e.g., fly ash, silica fume or granulated blast furnace slag) are required to mitigate ASR in RAC to achieve NAC’s levels.
- RA composed of non-reactive NA and non-reactive adhered mortar do not cause ASR in RAC; when RA are exclusively composed of high alkali reactive NA, ASR expansion in RAC increases with the increase of replacement ratio of NA with RA. A replacement level of 20% does not suggest a significant increase in mix reactivity.

Several doubts still without answer compromise the development of specifications for the use of RAC, promoting a sustainable future in construction. Conclusions are unanimous in that the study of RAC should always include the assessment of RA’s reactivity to alkalis. However, the heterogeneity that may exist in alkalis and reactive silica contents in RA makes this process more difficult to achieve.

ASR combat does not just mean eliminating the risk associated with the use of reactive aggregates, as this will have serious economic, environmental and sustainability implications. The main challenge for future research is to find ways to minimize the occurrence of the reaction even in the presence of reactive aggregates.

## Abbreviation

AMBT	accelerated mortar-bar test method
ASR	alkali-silica reaction
CMBT	concrete micro-bar test
CPT	concrete prism test
NAC	natural aggregate concrete
RAC	recycled aggregate concrete
CDW	construction and demolition waste
ITZ	interfacial transition zone
NA	natural aggregates
OC	original concrete
RA	recycled aggregates
